# Difference between observed and expected number of involved lymph nodes reflects the metastatic potential of breast cancer independent to intrinsic subtype

**DOI:** 10.18632/oncotarget.3647

**Published:** 2015-04-10

**Authors:** Ke-Da Yu, Yi-Zhou Jiang, Zhi-Ming Shao

**Affiliations:** ^1^ Department of Breast Surgery, Cancer Center and Cancer Institute, Shanghai Medical College, Fudan University, Shanghai, P.R. China

**Keywords:** breast cancer, metastatic potential, lymph node

## Abstract

**Purpose:**

Poor prognosis associated with metastasis in breast cancer patients highlights the critical need to develop an effective evaluation model for metastatic potential (MP). We hypothesized that MP could be also indicated by primary tumor size and involved lymph nodes (LNs).

**Methods:**

The expected number of involved LNs is defined as tumor size (cm) divided by 1.5. The effect of the surrogate for MP (defined as difference between the number of observed and expected involved LNs) on breast cancer-specific survival (BCSS) was investigated in the first cohort from SEER (*n* = 108,814). Validation was performed in another SEER cohort (*n* = 50,414) and a third cohort (*n* = 3,755).

**Results:**

MP is an independent predictor for BCSS in the overall population [hazard ratio (HR) for high MP: 2.92; 95% confidence interval (CI): 2.80–3.03] and in subgroups. The effect of surrogate for MP on survival was independent to intrinsic subtype, with adjusted HRs of 3.46 (95%CI, 2.02–5.93), 2.30 (95%CI, 1.64–3.24), 4.05 (95%CI, 2.85–5.76), and 1.45 (95%CI, 1.04–2.03) in luminal-A, luminal-B, triple-negative, and HER2-positive subtypes, respectively.

**Conclusion:**

Difference between the observed and expected number of involved LNs serves as an indicator for MP, which is independent to intrinsic subtype and could predict survival. Our findings need further validation.

## INTRODUCTION

Breast cancer mortality is typically linked with distant metastasis, which is the most lethal type of recurrence and practically undetectable at the time of diagnosis. [1, 2] The poor prognosis associated with metastasis highlights the critical need to better understand the biology of breast cancer metastasis and to develop an accurate evaluation model for metastatic potential (MP) after surgery for patients with operable disease.

With the development of intrinsic molecular subtype, clinicians use the surrogate of intrinsic subtype to determine MP. Intrinsic subtype correlates well with differences in prognosis, tumor aggressiveness, and response to available therapies. [3–5] Compared with luminal-like (luminal-A and luminal-B) tumors, HER2-positive and triple negative breast cancer (TNBC) are more likely to exhibit higher MP and are associated with markedly worse survival. Unfortunately, it is difficult for clinicians to discriminate different levels of MP within one subtype. Of note, emerging evidence demonstrates that very small tumors with extensive lymph node (LN) involvement can exhibit highly aggressive behavior compared with larger ones; [6] similarly, in the absence of LN involvement, large tumor size can be a surrogate for biologically indolent disease. [7] Consequently, the integrated understandings of LN involvement with tumor size might provide some useful information on tumor biology independent to intrinsic subtype.

We hypothesized that, within a certain subtype, the MP of breast cancer could be determined by the difference between the number of observed and expected involved LNs. First, we measured the quantitative ratio between tumor size and the number of involved LNs in the overall population. Using this ratio, we determined the expected number of involved LNs based on a given tumor size. The difference between the observed and expected numbers of involved LNs might serve as a surrogate for MP, which is proved closely associated with distance disease-free survival (DDFS) and breast cancer-specific survival (BCSS). To perform high-powered statistical analysis, we used the National Cancer Institute's Surveillance, Epidemiology and End Results (SEER) cancer database for testing and validation. We further validated our findings in another independent cohort from Fudan University Shanghai Cancer Center (FDUSCC).

## MATERIALS AND METHODS

### The first cohort from SEER for model establishment and the survival test

In the first cohort, we selected female patients with invasive breast cancer from the SEER database (released in Nov 2012) from Jan-1 1997 to Dec-31 2006. Patients diagnosed after 2006 were excluded to ensure an adequate follow-up time.

Initially, we identified 111,321 patients according to the following inclusion criteria: female, age of diagnosis between 18 and 74 years, surgical treatment with either mastectomy or breast-conserving surgery, AJCC TNM stages I-III, pathologically confirmed invasive ductal carcinoma, at least four axillary LNs dissected, unilateral cancer, known time of diagnosis, breast cancer as the first and only cancer diagnosis, known number of involved LNs, and known tumor size. The following information was also obtained if available: estrogen receptor (ER) and progesterone receptor (PgR) status, histological grade, race, and use of radiotherapy. A few cases with borderline values of ER/PgR were treated as positive because, according to current standards, ER and PgR status is considered positive if there are at least 1% positive nuclei. [8] Although SEER provides HER2 status from 2010 and the subtype of each case could be determined after that time, the follow-up time for survival is inadequate yet. SEER did not provide information on chemotherapy and endocrine therapy. There were very few cases with extreme values of tumor size (1.3% of cases were larger than 8.0 cm) and numbers of positive LNs (1.0% of cases had more than 18). To minimize the influence of extreme values, we excluded these cases. In total, 108,814 cases composed the first cohort.

The primary study outcome was BCSS. The cause of death was categorized as breast cancer-specific or non-breast cancer-related. BCSS was calculated from the date of diagnosis to the date of breast cancer death. Patients who died from other causes were censored at the date of death.

### The second cohort from SEER for survival validation

Using the criteria above, we selected 50,414 patients with invasive ductal breast cancer from SEER between Jan-1 1990 and Dec-31 1997 to validate the preliminary findings in the first cohort. Patients diagnosed before 1990 were excluded because of the lack of hormone receptor data. Because early cases might exhibit different distributions in tumor stage compared with those at present, [9] we did not use this set for linear regression to calculate the ratio of tumor size to the number of involved LNs.

### The third cohort from FDUSCC for survival validation

To further validate the findings from the SEER dataset, to determine a direct relationship between the MP category and distant metastasis, and especially to test the performance of MP category in a certain subtype, we used the data from 3,755 consecutive patients diagnosed with operable unilateral breast cancer between Jan-1 1998 and Dec-31 2006 at FDUSCC. This is a well-characterized series of patients, whose clinicopathologic and follow-up information were maintained on a prospective basis. [10] Patients' treatments were based on St. Gallen consensus. [11, 12] The cut-off for ER or PgR positivity was ≥ 10% of tumor cells with nuclear staining. Pathologic HER2 status was defined according to ASCO/CAP guidelines. [13] The primary treatment for all of these patients was surgery. Intrinsic subtypes (luminal-A, luminal-B, TNBC, and HER2-positive) were determined according to the clinicopathologic criteria recommended by the St Gallen panelists. [14] In brief, luminal-A is ER/PgR positive, HER2 negative, and Ki-67 low (< 14%); luminal-B is ER/PgR positive, and HER2 positive or Ki-67 high; TNBC is ER, PgR, and HER2 negative; HER2-positive is ER/PgR negative and HER2 positive. Because information on Ki-67 was not available in earlier cases, we used grade to capture cell proliferation, as suggested by von Minckwitz et al. [15]

The outcomes of interest were DDFS, which was calculated from the date of diagnosis to the date of first distant metastasis. To determine distant relapse events, isolated local recurrence was further followed until a metastasis event. The research protocol of this part of our study was reviewed and approved by the Ethical Committee and Institutional Review Board of FDUSCC. All patients provided written informed consent.

The basic characteristics of the patients in the three cohorts are presented in Table 1.

**Table 1:** Characteristics of patients from three cohorts

Characteristic	SEER set (1998–2006) *N* = 108,814		SEER set (1990–1997) *N* = 50,414		FDUSCC set (1998–2006) *N* = 3,755	
	No.	%	No.	%	No.	%
Median follow-up, months	92		174		79	
IQR	64–118		116–204		60–106	
Patient age, years						
≤ 50	42,344	38.9	18,992	37.7	1,849	49.2
> 50	66,470	61.1	31,422	62.3	1,906	50.8
Race						
White	87,162	80.4	41,788	83.1	0	0
Black	11,591	10.7	4,139	8.2	0	0
Others^*^	9,602	8.9	4,356	8.7	3,755	100.0
Unknown	459	-	131	-	-	-
Lymph node status						
Negative	59,207	54.4	32,531	64.5	1,981	52.8
Positive	49,607	45.6	17,883	35.5	1,774	47.2
Tumor size, mm						
0–20	65,315	60	32,832	65.1	1,623	43.2
21–50	38,628	35.5	15,999	31.7	1,957	52.1
≥ 51	4,871	4.5	1,583	3.1	175	4.7
Grade						
I	15,663	14.8	5,453	12.8	102	3.3
II	41,590	39.3	17,930	42	2,194	70
III or UD	48,597	45.9	19,276	45.2	838	26.7
Unknown	2,964	-	7,755	-	621	-
ER status						
Negative	26,839	27.5	11,578	27	1,192	37.5
Positive	70,637	72.5	31,227	73	1,985	62.5
Unknown	11,338	-	7,609	-	578	
PgR status						
Negative	35,609	37.1	14,225	34.1	1,366	43.3
Positive	60,475	62.9	27,439	65.9	1,786	56.7
Unknown	12730	-	8,750	-	603	-
HER2 status						
Negative	N.A.	N.A.	N.A.	N.A.	2,408	75.8
Positive	N.A.	N.A.	N.A.	N.A.	769	24.2
Unknown	N.A.	-	N.A.	-	578	-
Radiotherapy^&^						
No	47,653	45.3	26,794	54.4	2,273	61.6
Yes	57,654	54.7	22,416	45.6	1,417	38.4
Unknown	3,507	-	1,204	-	65	-
Adjuvant chemotherapy						
No	N.A.	N.A.	N.A.	N.A.	607	16.8
Yes	N.A.	N.A.	N.A.	N.A.	3,014	83.2
Unknown	N.A.	-	N.A.	-	134	-

Abbreviations: ER, estrogen receptor; FDUSCC, Fudan University Shanghai Cancer Center; HER2, human epidermal growth factor receptor-2; IQR, inter-quartile range (from 75^th^ percentile to the 25^th^ percentile); N.A., not applicable; PgR, progesterone receptor; SEER, Surveillance, Epidemiology and End-Results registry; UD, undifferentiated

* Including American Indian, Alaska Native, Asian, and Pacific Islander

& In FDUSCC set, radiotherapy is in adjuvant setting.

### Statistics

The median follow-up times were 92, 174, and 79 months for the first, second, and third cohort, respectively; we therefore reported the 8-year, 15-year, and 6-year rates of survival, respectively.

In the first set, we performed an exploratory analysis of the relationship between tumor size and the number of involved LNs (both as continuous variables). Because we could not rule out a nonlinear relationship, we regressed the number of involved LNs on tumor size using nonparametric regression based on either locally weighted scatterplot smoothing (LOWESS; the command “lowess” in Stata) [16] or Kernel-weighted local polynomial smoothing method (the command “lpoly” in Stata). [17] If a linear relationship was revealed, linear regression was employed to determine the quantitative relationship between tumor size and the observed number of involved LNs. Linear regression was performed with or without adjustments for other clinicopathologic factors. By this procedure, the expected (predicted) number of positive LNs of each case could be calculated. The continuous variable of the difference between the observed and expected number of involved LNs was used as the surrogate for MP. The nonlinear effect of continuous values of the surrogate of MP on BCSS was assessed using a B-spline transformation with evenly spaced knots. High MP was defined as a difference between the observed number and expected number (the former minus the latter) greater than or equal to 1; otherwise, the MP was considered to be low/normal.

Survival curves were constructed using the Kaplan-Meier method, and the univariate survival difference was determined by log-rank test. Survival time was estimated using life-table method. Adjusted hazard ratios (HR) with 95% confidence interval (CI) were calculated using the Cox proportional hazards models. All of the statistical analyses were performed using Stata v.10.0 (Stata Corporation, College Station, TX). Two-sided *P* < 0.05 was considered statistically significant.

## RESULTS

### A linear relationship between tumor size and the number of involved axillary LNs in the first cohort from SEER

**Figure 1:**
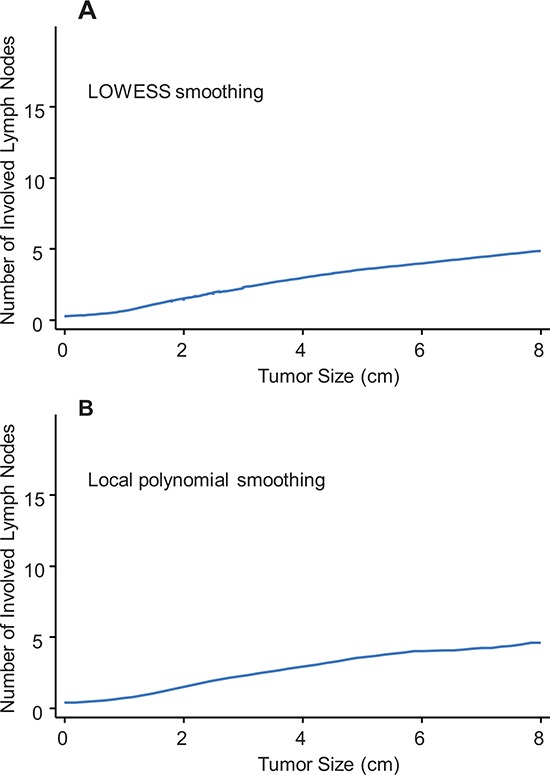
**Relation between tumor size and number of involved lymph nodes based on univariate nonparametric smoothing method using LOWESS A.** or local polynomial smoothing **B.**

Clinicians use the TNM staging system, which provides a description of the extent and spread of a tumor, to determine the severity of disease. Specifically, the TNM stage is determined by tumor size, LN involvement, and whether cancer has metastasized. Generally, larger tumors are positively associated with greater numbers of involved LNs. To explore the potential relationship (either linear or nonlinear) between tumor size and the number of involved LNs, the continuous number of positive LNs was regressed on the continuous tumor size (cm) using LOWESS (Figure 1A) or the local polynomial smoothing method (Figure 1B). Both methods consistently suggested a monotonic linear relationship between tumor size and LN involvement in the overall population. Subsequently, linear regression was used to predict the number of positive LNs according to tumor size. The regression formula was Y (number of involved LNs) = 0.70* × (tumor size) + 0.09 (*P* < 0.001 for the coefficient, *P* < 0.001 for the regression model), indicating that the number of involved LNs increased by 0.70 for each centimeter increase in size. In other words, for each LN involved, tumor size should increase by 1.43-cm. After adjusting for age, year of diagnosis, race, grade, and ER status, the coefficient was 1.66 (*P* < 0.001), i.e., when tumor size progressed by 1.52-cm, one more LN would be involved. Notably, race had no effect on the correlation between tumor size and the number of involved LNs (*P* = 0.29). To achieve feasible utility, we use the approximate value of 1.50 to calculate the expected number of involved LNs for a given tumor size. The agreement between the approximate value of 1.50 and the original value of 1.52 was measured, and the Cohen's Kappa coefficient was 0.95, indicating perfect agreement. [18] Therefore, the ratio of 1.5 was an acceptable value for feasible use. We calculated the expected number of involved LNs for each case. For instance, a patient with a 1.5-cm breast tumor theoretically had 1 (1.5/1.5) positive node; when the tumor further progressed to 3.0-cm with no treatment, theoretically 2 (3.0/1.5) regional LNs should be involved.

### The difference between the observed number and the expected number of involved LNs serves as a significant surrogate for MP

**Figure 2:**
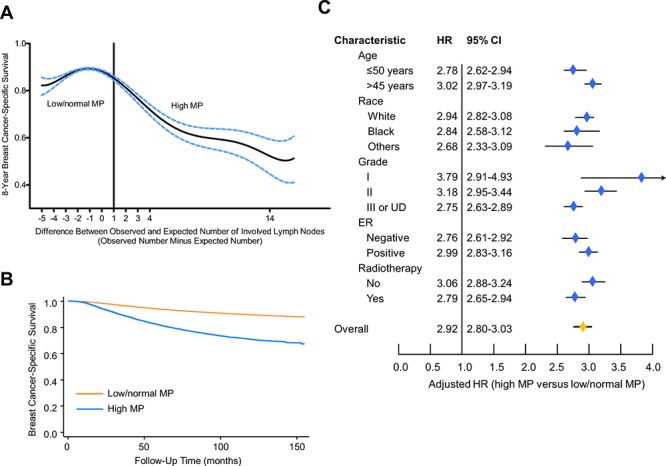
Effect of surrogates for metastastic potential (MP) on breast cancer-specific survival (BCSS) **A.** Relationship between the continuous values of MP (determined by the difference between the number of observed and expected involved LNs) and 8-year BCSS. We chose a value of 1 as the cutoff between high and low/normal MP. **B.** Kaplan-Meier curves for patients by categorical MP. **C.** Forest plot of multivariate analysis. The hazard ratios (HRs) with 95% confidence intervals (CI) for the patients with high MP, compared with those with low/normal MP, were assessed using the Cox regression model by adjusting for all other prognostic factors listed. The diamond denotes the HR of each subgroup. An HR > 1.0 indicates higher risk for breast cancer-specific mortality in the high MP group and vice versa.

Because the ratio of tumor size to the number of involved LNs was approximate 1.5 in the overall population, we calculated the expected number according to tumor size for each case. The relationship between the continuous value for numerical difference (observed value minus expected value) and 8-year BCSS is illustrated in Figure 2A, which reveals a pattern of comparable 8-year BCSS when the value for the numerical difference is less than 1, with a peak at the value of −1; when this value is greater than 1, the increasing value is related to remarkably decreasing BCSS. Accordingly, we determined that, if a patient's numerical difference (observed number minus expected value) was greater than or equal to 1, she was assigned to the high MP group; otherwise, the patient was assigned to the low/normal MP group.

We then studied the prognostic value of categorical MP. In univariate analysis, a survival difference was noted between the high MP and low/normal MP groups, with 8-year BCSS rates of 74.1% (95% CI, 73.4–74.7%) and 91.1% (95% CI, 90.9–91.3%), respectively (Figure 2B). The unadjusted HR for high MP was 3.20 (95% CI, 3.09–3.32) relative to low/normal MP (Table 2). In multivariate analysis using the Cox model, after adjustment for other prognostic indicators, the HR of high MP was 2.92 (95% CI, 2.80–3.03; Table 2). Moreover, the prognostic significance of this surrogate of MP persisted in each subgroup stratified by other prognostic factors (Figure 2C), even in each subgroup stratified by tumor size (Table 2).

**Table 2:** Univariate and multivariate analysis of survival in three cohorts

	MP	BCSS in SEER set (1998–2006)					BCSS in SEER set (1990–1997)					DDFS in FDUSCC set (1998–2006)				
		Univariate		Multivariate			Univariate		Multivariate			Univariate		Multivariate		
		8-yr rate	P^*^	HR^#^	95% CI	*P*	15-yr rate	P^*^	HR^#^	95% CI	*P*	6-yr rate	P^*^	HR^&^	95% CI	*P*
**Overall**																
	Low/normal	91.1	-	1.00	Ref.	-	83.1	-	1.00	Ref.	-	91.0	-	1.00	Ref.	-
	High	74.1	< 0.001	2.92	2.80–3.03	< 0.001	51.8	< 0.001	3.25	3.11–3.41	< 0.001	72.3	< 0.001	2.60	2.11–3.18	< 0.001
**Tumor Size**																
0–20 mm	Low/normal	95.3	-	1.00	Ref.	-	88.7	-	1.00	Ref.	-	93.3	-	1.00	Ref.	-
	High	84.6	< 0.001	2.93	2.73–3.15	< 0.001	63.2	< 0.001	3.58	3.33–3.86	< 0.001	80.0	< 0.001	2.58	1.76–3.78	< 0.001
21–50 mm	Low/normal	84.5	-	1.00	Ref.	-	71.3	-	1.00	Ref.	-	90.0	-	1.00	Ref.	-
	High	67.7	< 0.001	2.43	2.31–2.57	< 0.001	44.0	< 0.001	2.39	2.24–2.54	< 0.001	73.1	< 0.001	2.29	1.75–3.01	< 0.001
≥ 51 mm	Low/normal	74.2	-	1.00	Ref.	-	61.8	-	1.00	Ref.	-	72.1	-	1.00	Ref.	-
	High	51.3	< 0.001	2.45	2.19–2.75	< 0.001	28.7	< 0.001	2.62	2.20–3.10	< 0.001	45.8	< 0.001	1.99	1.09–3.65	0.03
**Subtype**																
Luminal A	Low/normal	N.A.	-	N.A.	N.A.	-	N.A.	-	N.A.	N.A.	-	96.9	-	1.00	Ref.	-
	High	N.A.	N.A.	N.A.	N.A.	N.A.	N.A.	N.A.	N.A.	N.A.	N.A.	86.2	< 0.001	3.46	2.02–5.93	< 0.001
Luminal B	Low/normal	N.A.	-	N.A.	N.A.	-	N.A.	-	N.A.	N.A.	-	86.6	-	1.00	Ref.	-
	High	N.A.	N.A.	N.A.	N.A.	N.A.	N.A.	N.A.	N.A.	N.A.	N.A.	62.9	< 0.001	2.30	1.64–3.24	< 0.001
TNBC	Low/normal	N.A.	-	N.A.	N.A.	-	N.A.	-	N.A.	N.A.	-	81.5	-	1.00	Ref.	-
	High	N.A.	N.A.	N.A.	N.A.	N.A.	N.A.	N.A.	N.A.	N.A.	N.A.	51.2	< 0.001	4.05	2.85–5.76	< 0.001
HER2+	Low/normal	N.A.	-	N.A.	N.A.	-	N.A.	-	N.A.	N.A.	-	79.0	-	1.00	Ref.	-
	High	N.A.	N.A.	N.A.	N.A.	N.A.	N.A.	N.A.	N.A.	N.A.	N.A.	69.1	0.05	1.45	1.04–2.03	0.03

Abbreviations: BCSS, breast cancer-specific survival; CI, confidence interval; DDFS, distant disease free survival; Ref., reference; FDUSCC, Fudan University Shanghai Cancer Center; HER2, human epidermal growth factor receptor-2; HR, hazard ratio; MP, metastatic potential; N.A., not applicable; SEER, Surveillance, Epidemiology and End-Results registry; TNBC, triple negative breast cancer

* log-rank test

# in SEER sets, HR was adjusted for age at diagnosis, race, grade, ER, and radiotherapy. Cox regression model (method: backward, likelihood ratio) is employed to calculate HR.

& in FDUSCC set, HR was adjusted for age at diagnosis, grade, ER, HER2, adjuvant chemotherapy, and adjuvant radiotherapy for the overall population and subgroups stratified by tumor size. In subgroups according to intrinsic subtype, HR was adjusted for age at diagnosis, grade, adjuvant chemotherapy, and adjuvant radiotherapy.

### Validation of MP in the second cohort from SEER

**Figure 3:**
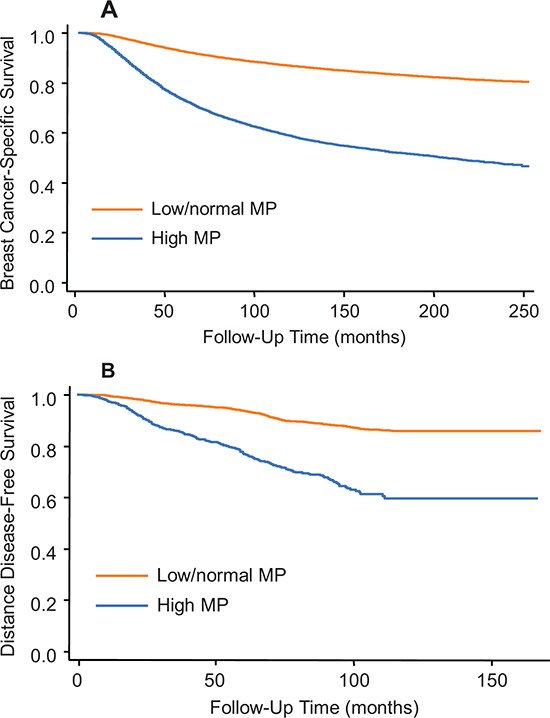
Kaplan-Meier curves for the second cohort from SEER **A.** and the third cohort from FDUSCC **B.** stratified by categorical MP, respectively. P values for Kaplan-Meier curves in both plots are < 0.0001 by log-rank test.

To validate the prognostic effect of the surrogate of MP on breast cancer survival, we chose an additional 50,414 cases from the SEER registry from 1990 to 1997. The high and low/normal MP groups demonstrated 15-year BCSS rates of 51.8% (95 CI, 50.7–52.9%) and 83.1% (95% CI, 82.7–83.5%)(Figure 3A), respectively, with an unadjusted HR of 3.62 (95% CI, 3.48–3.76) and an adjusted HR of 3.25 (95% CI, 3.11–3.41) (Table 2).

### Validation of MP in the third cohort from FDUSCC

In this cohort, the endpoint of survival analysis was distant metastasis. Consistent with observations in SEER, higher MP was associated with an increased risk of distant metastasis (Kaplan-Meier curves shown in Figure 3B; adjusted HR for DDFS was 2.60; 95% CI, 2.11–3.18). Because information concerning the intrinsic subtype was available in our dataset, we analyzed each subtype separately and found that MP was a prognostic factor independent of the subtype (Table 2). In luminal-A, luminal-B, TNBC, and HER2-positive subtypes, the high MP group had an adjusted HR of 3.46 (95% CI, 2.02–5.93), 2.30 (95% CI, 1.64–3.24), 4.05 (95% CI, 2.85–5.76), and 1.45 (95% CI, 1.04–2.03), respectively, when compared with low MP. The value of adjusted HR for HER2-positive group was relative lower than those in other subtypes, which might be because about 50% cases of HER2-positive cases received adjuvant trastuzumab.

## DISCUSSION

In the present study, we sought to determine whether there was a clinicopathologic surrogate for the MP of breast cancer. We hypothesized that the difference between the expected number of involved LNs according to tumor size and the actual number of involved LNs might serve as an indicator for MP and thus could predict survival. Using a large-population cohort from SEER, we identified a linear relationship between tumor size and the number of involved LNs, and we subsequently established a prediction model for the expected number of involved LNs at a given tumor size. By this procedure, we classified patients into a high MP or low/normal MP group according to the difference between the observed and expected number. After adjustment for other prognostic factors, patients with high MP had a higher likelihood of death from breast cancer compared with those with low/normal MP. The prognostic effect was subsequently successfully validated in other two cohorts and proved to be independent to intrinsic subtypes. To the best of our knowledge, the influence of differences between the observed and expected numbers of involved LNs on MP has not yet been proposed.

The conventional view of cancer spread is that cancer gains metastatic ability through the accumulation of mutations as the tumor grows to a large size. [3] In overall population, it is an indisputable fact that size of the primary tumor is positively related to LN involvement, suggesting that MP evolves as the tumor grows. [19] However, there is increasing awareness of tumor biology in predicting patient outcome. A growing body of literature demonstrates that distant metastasis could be, to some extent, determined by the intrinsic biology of breast cancer rather than the local disease severity. [20, 21] Clinically, the abnormal relationship between tumor size and involved LNs suggests varied tumor biology. [6, 7] Currently, there are limited numbers of clinicopathologic markers to assess the MP of breast cancer. Conventional TNM staging does not work well to determine MP, and the intrinsic subtype cannot further discern MP subgroups within one subtype.

We developed a quantitative marker, the numerical difference between the observed and expected numbers of involved LNs, to reflect different levels of MP. The patients with values less than −3 (mainly T_3_ tumor with negative nodes) had slightly lower BCSS compared with those with value of −3 to 1. In contrast, once the value exceeded 1, the survival curve began to decrease in a monotonic pattern. For a feasible evaluation, we arbitrarily divided patients into two classes, high or low/normal MP with a cutoff value at a numerical difference of 1. It should be noted that our model might have limited predictive capability in larger tumors, as tumors with numerical differences of −5 to −3 exhibit comparably poor survival relative to tumors exhibiting values of 1 to 3. In contrast, our model is excellent for the personalized evaluation of MP in T_1–2_ tumors with extensive LN involvement. For instance, a patient with a 1-cm tumor and 10 positive LNs and another patient with a 2.5 cm tumor and 10 positive LNs share the same pathological TNM stage. However, the value for the numerical difference (observed number minus expected number) differs. Our model predicts better survival for the patient with the 2.5 cm tumor and 10 positive LNs. In SEER set between 1998 and 2006, we identified 12 cases with 1-cm tumors and 10 involved LNs and 70 cases with 2.5-cm tumors and 10 involved LNs. The actual survival outcomes demonstrate that 7 of 12 (58%) and 21 of 70 (30%) died from breast cancer in the former and latter groups, respectively, in accordance with the predicted MP levels.

Taken together, the surrogate marker of MP derived from tumor size and number of involved LNs provides us a simple but effective tool to determine the potential for distant metastasis. Notably, the linear relationship between tumor size and the number of involved LNs is independent of race, implying that our findings, originally from a western population, could be extrapolated to Asian and other populations. Indeed, successful validation in a Chinese population supports this assumption and warrants the worldwide use of this model.

Our study had several limitations. First, the SEER database lacks several important variables such as HER2 status, adjuvant chemotherapy, and recurrence type. We could not adjust for more confounding factors, nor could we directly investigate the effect of surrogates of MP on DDFS. Second, our study was limited to invasive ductal histology; thus, our findings cannot be extrapolated to other histology types. Moreover, our model should be used with caution for cases with large tumor sizes. The current model based on tumor size and involved number of nodes seems to have limited capability to predict the survival of very larger tumor with negative nodes disease. Although the potential for distant metastasis may not be large enough for large tumors with negative nodes, [7] the local tumor burden is heavy, and the likelihood of recurrence would be much higher, which could negatively affect survival. [22] Despite these limitations, our data represent the most robust evaluation of the effect of tumor size and the number of involved LNs on MP in breast cancer.

In conclusion, our study reveals that differences between the expected number of involved LNs according to tumor size and the observed number of involved LNs might serve as an indicator for MP, and this surrogate for MP could predict survival. We introduced a simple but effective tool to determine breast cancer MP by comprehensively understanding the relationship between tumor size and the number of involved LNs. A deeper understanding of the biology of breast cancer using common clinicopathologic factor-based tools would certainly help clinicians to predict distant metastasis and provide personalized systemic therapies.
